# Exploring subcolony differences in foraging and reproductive success: the influence of environmental conditions on a central place foraging seabird

**DOI:** 10.1098/rsos.220362

**Published:** 2023-06-28

**Authors:** Jessica Pulvirenti, Richard D. Reina, Andre Chiaradia

**Affiliations:** ^1^ School of Biological Sciences, Monash University, Clayton, Victoria 3800, Australia; ^2^ Conservation Department, Phillip Island Nature Parks, PO Box 97, Cowes, Victoria 3922, Australia

**Keywords:** segregation, seabird subcolonies, foraging success, reproductive success, sea surface temperature, *Eudyptula minor*

## Abstract

While differences in foraging and reproductive success are well studied between seabird colonies, they are less understood at a smaller subcolony scale. Working with little penguins (*Eudyptula minor*) at Phillip Island, Australia, we used an automated penguin monitoring system and performed regular nest checks at two subcolonies situated 2 km apart during the 2015/2016 breeding seasons. We examined whether foraging and reproductive success differed between subcolonies. We used satellite data to examine how sea surface temperature, as environmental pressure, in the foraging regions from each subcolony influenced their foraging performance. In the pre-laying and incubation breeding stages, the birds from one subcolony had a lower foraging success than birds from the other. However, this pattern was reversed between the subcolonies in the guard and post-guard stages. Breeding success data from the two subcolonies from 2004–2018 showed that reproductive success and mean egg-laying had a negative relationship with sea surface temperature. We highlighted that variation in foraging and reproductive success can arise in subcolonies, likely due to different responses to environmental conditions and prey availability. Differences at the subcolony level can help refine, develop and improve appropriate species management plans for conserving a range of colonial central place seabirds.

## 1. Introduction

Limitation in space and food resources is one of the most critical factors contributing to competition in colonial central place foraging seabird populations [1]. Although coloniality is an advantageous adaptation commonly found in seabirds, closely associating with conspecifics can result in density-dependent competition [2–5]. During the breeding season, parent birds are constrained to a central place, which can increase the competition between conspecifics in the area [6–9]. Density-dependent competition affects the growth rate of a colony, with an increasing colony size creating a decline in growth rate [1,10].

The ecological niche theory proposes that resources must be segregated for morphologically similar species to coexist, particularly in space, time or diet [11,12]. When there is spatial segregation of foraging and breeding sites, between colonies or within colonies, the competition for resources can decrease [13–16]. Subcolonies exist when spatial segregation occurs within a colony. These subcolonies may experience a higher level of competition than exists with between-colony segregation, due to conspecifics using more similar resources [17]. Increased competition for resources is also likely to be more evident when there is a higher constraint, typically low prey availability [18]. Other than the competition for resources, factors such as age, sex, breeding status and predation can also lead to the spatial segregation of foraging and breeding sites [19–21].

Understanding how the environment influences seabirds' foraging and reproductive success is imperative [22]. In particular, sea surface temperature (SST) is a good predictor of reproductive success, prey abundance and prey distribution [23–25]. Prey abundance is generally higher during the warmer months when many seabirds use this as a signal to start breeding [24]. However, foraging opportunities can be limited when SST is above the average as prey, such as clupeids, have a low thermal tolerance in these conditions [23,26,27]. Prey distribution can be influenced by stratification in the water column and by differences in SST which can result from factors such as water currents, wind, and tide [28–31]. As SST is quite conservative in space and time, small changes can greatly alter prey abundance and distribution. Even within small regions, these differences are likely to occur at both the colony and the subcolony level [20,32,33].

When prey is limited, seabirds can alternate between short and long foraging trips, prey on more energy-dense species, forage in different areas, and increase their foraging range [34–36]. An animal under poor prey availability and environmental conditions may prioritize their fitness over their chicks’ and abandon a breeding attempt [37,38]. Many studies have investigated the impact of environmental conditions and variation in foraging and reproductive success between seabird colonies (e.g. [16,39–41]). However, information is scarce on these factors at a finer scale within a colony [14,42,43]. Many seabird studies investigate one specific area of a colony due to ease of data collection or technological limitations [29,44,45]. However, if there are differences in foraging and reproductive success within a colony it would identify the need for future studies on central place foragers to be investigated at this finer scale to inform effective conservation management strategies.

The little penguin (*Eudyptula minor*) is the smallest penguin species and has one of the shortest foraging ranges among seabirds, approximately 20 km or less from their colony during the breeding season [7,46]. This short foraging range poses a challenge for the parents to meet the energy requirements of their chicks and themselves [7]. Within the breeding season, there are four breeding stages; pre-laying (30 days), incubation (35 days), guard (15 days) and post-guard (42 days on average) [47]. One of the largest colonies of little penguin species at Phillip Island, Australia has an estimated 28 000 to 32 000 individuals [48]. Due to intra-specific competition, the colony has undergone a natural division into subcolonies, each occupying different foraging and breeding sites, with little spatial overlap [1,10,43]. Throughout the breeding season, the spatial foraging segregation of these subcolonies can vary from partial to complete. By contrast, if they were separate colonies, there would consistently be complete segregation [43].

We used two subcolonies of little penguin on Phillip Island as a model system to determine their variation in foraging and reproductive success. Given the spatial segregation of the subcolonies at Phillip Island, we further investigated the differences in reproductive success and whether the timing of breeding of each subcolony was influenced by the environmental conditions at each subcolony's foraging area. By expanding on the data set used by [43], we predicted that (1) there would still be sub-colony differences in adult body mass change per day of a foraging trip and also in foraging trip duration over all four breeding stages, (2) there would still be differences in the reproductive success and also in mean laying dates of each subcolony as a result of these foraging differences and (3) that there would be a difference in the sea surface temperature at each subcolony's foraging site which would be an indicator of thermal conditions in the area and this difference would influence the reproductive success and mean laying date of each subcolony.

## 2. Material and methods

### 2.1. Study site

**Figure 1.**
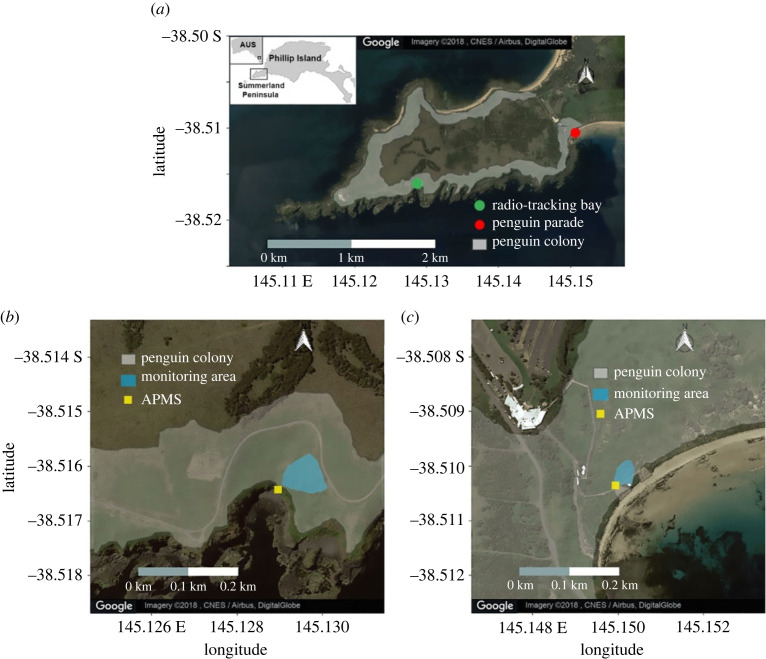
Satellite images showing (*a*) the distribution of the penguin colony around the Summerland Peninsula, Phillip Island and the location of each study site. Also shown is the area where the monitored nests are and the location of the automated penguin monitoring system (APMS) at (*b*) Radio-tracking Bay and (*c*) Penguin Parade. (Figure taken from [43]).

We investigated spatial segregation of foraging and breeding sites between two little penguin subcolonies in the mega-colony of 32 000 birds at Phillip Island, Victoria, Australia (38°31′S, 145°09′E) [48]. The two subcolonies are located approximately 2 km apart, with one subcolony at Radio-tracking Bay and the other at Penguin Parade, with approximately 100–150 breeding pairs in each (figure 1). The breeding season for little penguins occurs in the Austral summer, usually between September and February [47]. A breeding pair can lay 1–2 eggs per clutch and have up to three clutches if it is a successful breeding season, but usually only lay one clutch at Phillip Island [47].

### 2.2. Foraging success and automated data collection

Foraging success was indicated by foraging trip duration and body mass change per day. Foraging trip duration is the number of days an individual spent at sea in one trip. It was calculated by: Day of the year returning to the subcolony – Day of the year leaving the subcolony. Body mass change per day was defined as body mass, to the nearest 1 g, gained or lost by an individual for each day of a foraging trip. It was calculated by: (Mass (g) returning to the subcolony – Mass (g) leaving the subcolony)/Foraging trip duration (days). To determine how foraging success differed between subcolonies, the analytical models included foraging trip duration and body mass change as response variables, and breeding season, sex, age, and location (i.e. foraging site) as predictor variables, with a random effect of transponder number to account for individual differences. Breeding season, sex and age were included in the models because they may also influence the foraging success of a subcolony [20,21].

The data that were used to determine foraging success were collected from the 2015 and 2016 breeding seasons. Each subcolony's breeding site had an automated penguin monitoring system (APMS) installed to detect the movement of birds in and out of the subcolony (figure 1). The APMS at each site was located on the main penguin pathway into the subcolony [49]. Each time the penguin entered and left the colony, it passed through the APMS and the date, time, penguin body mass (g) and ID were recorded [47]. The APMS records when the penguins enter and leave the colony on successive trips (from which foraging trip duration can be calculated) and the mass to the nearest gram before and after each successive foraging trip (from which change in body mass can be calculated). Each APMS was checked and the weighing platform calibrated weekly.

Each record from the APMS was marked as either ‘IN’ or ‘OUT’ to indicate whether the penguin was entering or leaving the colony, respectively. If an individual was recorded in the 12 h before UTC + 15 : 00, the record was marked ‘IN’ and if recorded in the 12 h after, was marked ‘OUT’ to correspond with their typical foraging patterns [47]. Individuals sometimes do not pass through the APMS when entering or leaving the colony, so there were records with consecutive ‘IN’ or consecutive ‘OUT’ values. To account for this, any records greater than 12 h apart with consecutive ‘IN’ or ‘OUT” values had a record with the opposite value added at an equal time between the two consecutive values. The records with interpolated data were only used to analyse foraging trip duration and not used for body mass change because foraging trip duration is generally much more consistent in each breeding stage, while body mass change can be more variable and not as well predicted [29]. Foraging trip durations of ≥15 days were considered outliers and removed [47]. Body mass change per day was limited to between –50 g and 420 g as changes outside this range were also considered outliers [50].

### 2.3. Reproductive success and nest monitoring

Reproductive success was measured as the number of chicks that had fledged per breeding pair in a single season. Chicks fledged per pair was calculated from the number of chicks fledged in a breeding season divided by the number of breeding females in the breeding season.

We monitored 100 nests at Radio-tracking Bay and Penguin Parade subcolonies throughout the breeding seasons from 2004 to 2018 to collect penguin breeding data. The subcolony at the Penguin Parade was checked three times per week, while the subcolony at Radio-tracking Bay was checked once every two weeks, except for the breeding seasons in 2015 and 2016 when it was checked weekly [43]. Sites were checked at different intervals due to the logistics of accessing different parts of the colony and accommodating various studies. The intervals were still much less than the time the birds spent in any of the breeding stages, so there was little impact on data quality. Penguin Parade had artificial nestboxes and natural burrows, and Radio-tracking Bay had natural burrows only.

Each nest was checked for occupancy at the frequency described for the presence of adults, chicks and eggs. This provided each nest's egg-laying dates, hatching dates and fledging dates. Chicks that were fully feathered and were aged greater than 40 days when last encountered were considered to have fledged [34]. Birds were permanently identified with a passive identification transponder (TIRIS, Texas Instruments, USA, Trovan, Australia and Allflex, Australia) read by a handheld scanner. Newly encountered adults and chicks had a transponder inserted subcutaneously between the scapulae for subsequent identification (see details in [47]). Adults without ID were tagged with a transponder when encountered more than once in the same nest and considered three years old when encountered [51]. Chicks were marked at seven weeks old. The sex of each penguin was determined by bill measurements [52].

### 2.4. Comparing reproductive success, the timing of breeding and environmental conditions

To determine how reproductive success, the timing of breeding and environmental conditions differed between subcolonies, the number of chicks fledged per pair, the mean laying date and the SST in the foraging sites of each subcolony, respectively, were compared between the subcolonies. To determine the impact of environmental conditions, we compared the number of chicks fledged per pair and the mean egg-laying date for each subcolony with the mean value of SST in the foraging area of each subcolony.

When SST in the waters surrounding the Phillip Island mega-colony increases after the Austral winter, the penguins use this as a signal to start breeding [24]. The lag time between this increase in temperature and the mean laying date is usually about seven weeks [24]. Therefore, SST for each subcolony foraging area was calculated as the average daily value between the 6th and 7th week before the mean laying date for the birds at Penguin Parade from the 2004–2018 breeding seasons [24].

Sea surface temperature was obtained through satellite data from Copernicus Marine Environment Monitoring Service [53]. The boundaries of the foraging areas of each subcolony that were used to obtain SST were determined by Sánchez *et al*. [43] where the birds from each subcolony were tracked at sea and their foraging areas located. Daily SST was collected from 2004 to 2018 at a spatial resolution of 0.083°. The mean value of SST of each foraging area of birds from Radio-tracking Bay was recorded within 38′S and 40′S and 144′E and 145′E, and from Penguin Parade was recorded within 38′S and 40′S and 145′E and 146′E [43].

### 2.5. Statistical methods

All statistical analyses were conducted using R 3.5.3 [54] and RStudio 1.0.136 [55]. Foraging trip duration and body mass change were analysed for each breeding stage from the 2015 and 2016 breeding seasons, as these were the only years of breeding stage data available. For this study, only individuals with a transponder number monitored for reproductive success at each subcolony were included in the foraging data.

**Figure 2.**
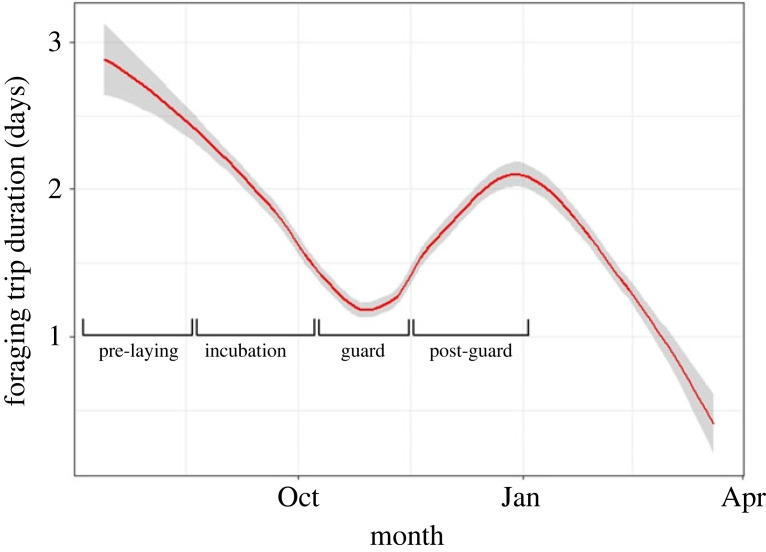
Foraging trip duration (days) of the Little Penguin over the 2016 breeding season on the Summerland Peninsula at Phillip Island, Australia. Each breeding stage is shown: pre-laying, incubation, guard and post-guard. The plot was smoothed using a local polynomial regression fitting to reduce noise. The grey shaded area indicates the 95% confidence intervals.

We used generalized linear mixed-effects models to detect any differences in foraging trip duration between breeding sites (Family = Poisson, Link function = Log). However, the data were highly skewed, so we used a mixed effects cox proportional survival analysis from the R package ‘coxme’ [56]. The 'survival' goal was defined as a penguin returning from a one-day trip versus 2+ day foraging trips. This analysis was performed for each breeding stage because there are different parental requirements for trip duration and the need to feed chicks; therefore, they can be considered independent (figure 2) [47].

The assumption of proportional hazards (1 trip versus 2+ trips) was checked using the R package ‘survival’ [57]. An interaction between location and foraging trip duration was included only in the model for the incubation breeding stage to meet assumptions. No other interactions were observed between the predictor variables (i.e. breeding season, sex, age and location), so they were removed from the model. The full model for the pre-laying, guard and post-guard breeding stages was: Foraging trip duration ∼ Season + Sex + Age + Location + (1| transponder). The full model for the incubation breeding stage was: Foraging trip duration ∼ Season + Sex + Age + Location + Location: Foraging trip duration + (1| transponder). Significance was set at less than 0.05.

**Figure 3.**
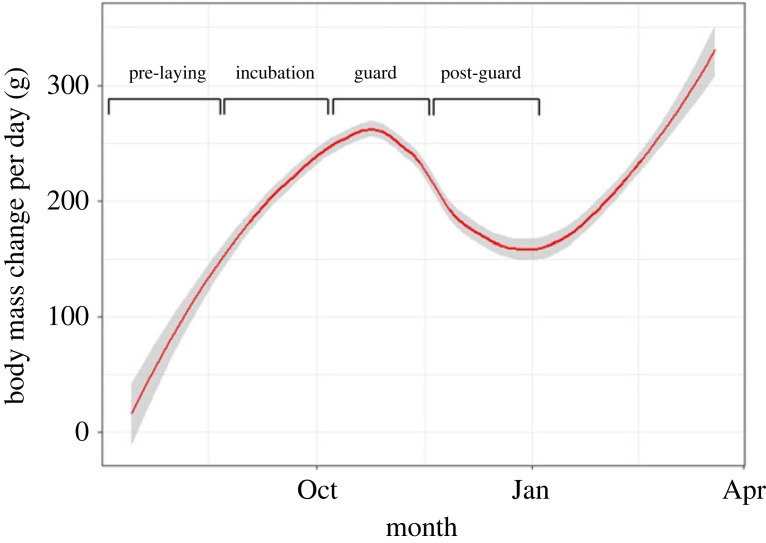
Body mass change per day (g) of the Little Penguin over the 2016 breeding season on the Summerland Peninsula at Phillip Island, Australia. Each breeding stage is shown: pre-laying, incubation, guard and post-guard. The plot was smoothed using a local polynomial regression fitting to reduce noise. The grey shaded area indicates the 95% confidence intervals.

To determine the relationship between body mass change and breeding site, a linear mixed-effects model was used to analyse body mass change from the R package ‘lmerTest’ [58]. The assumptions that the residuals were normally distributed, independent, and had equal variances were checked via residual plots and were met. This analysis was performed for each breeding stage as there are different parental requirements in each stage, and therefore body mass will differ in each stage (figure 3) [47]. The full model for each breeding stage was: body mass change per day ∼ Season + Sex + Age + Location + (1| transponder). No interactions were observed between the predictor variables (i.e. breeding season, sex, age and location) so they were removed from the model. Significance was set at less than 0.05.

Model selection was performed for foraging trip duration and body mass change using the ‘dredge’ function from the R package ‘MuMIn’ [59]. The ‘dredge’ function generates a list of models with different combinations of predictor variables. Akaike's information criterion was used to select the best-fit model [60]. The best-fit models had the sum of Akaike weights ≥0.9. From these models, the predictor variables' relative importance weights (RIW) were calculated by the sum of Akaike weights of the best-fit models where the predictor variable was present. The intervals to determine effect were: RIW ≥ 0.9 = strong effect, 0.6 ≥ moderate effect less than 0.9, 0.5 ≥ very weak effect less than 0.6, RIW less than 0.5 = no effect. For interactions, RIW > 0.7 = strong effect and RIW > 0.5 = moderate effect [61].

Paired t-tests were performed by pairing each subcolony, Penguin Parade and Radio-tracking Bay, for the breeding seasons from 2004 to 2018. A paired *t*-test was performed for the response variables; chicks fledged per pair, mean laying date and SST. The data for chicks fledged per pair from 2004–2015 was sourced from Sánchez *et al*. [43]. The data from Sánchez *et al*. [43] were tested using further years of data to compare by year with mean laying date and SST data. The normality of data was checked using Shapiro-Wilks normality tests, and equal variances were checked using Bartlett tests of homogeneity of variances. All assumptions were met. Significance was set at less than 0.05.

## 3. Results

### 3.1. Effect of location on foraging trip duration and body mass change

**Figure 4.**
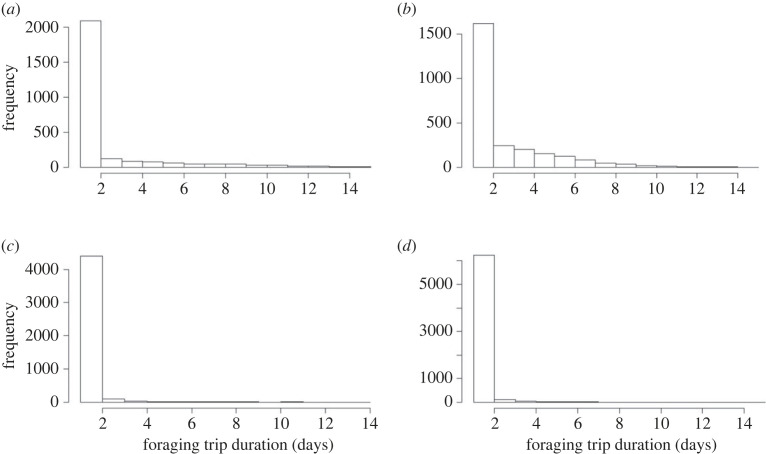
Histograms showing the frequency of foraging trip duration (days) for birds at both locations for the 2015 and 2016 breeding seasons for the (*a*) pre-laying, (*b*) incubation, (*c*) guard and (*d*) post-guard breeding stages.

Over the two breeding seasons for both locations, 16 221 penguin crossing records from the APMS were used to analyse foraging trip duration and 5337 crossings were used to analyse body mass change (table 1). Foraging trip duration of one day had the highest frequency in all breeding stages (69% of records in the pre-laying stage, 53% of records in the incubation stage, 92% of records in the guard stage, 87% of records in the post-guard stage) (figure 4).

**Table 1.** The total number of records from the automated penguin monitoring system that were used for the analysis and mean foraging trip duration and body mass change per day for the birds at each location in each breeding stage from the 2015 and 2016 breeding seasons.

breeding stage	foraging trip duration			body mass change		
	total number of records	penguin parade (days ± s.e.)	radio-tracking bay (days ± s.e.)	total number of records	penguin parade (g ± s.e.)	radio-tracking bay (g ± s.e.)
pre-laying	2665	2.32 ± 0.06	2.05 ± 0.08	778	125.43 ± 4.91	160.68 ± 6.17
incubation	2536	2.75 ± 0.06	2.32 ± 0.08	917	131.25 ± 4.42	159.60 ± 5.62
guard	4570	1.16 ± 0.01	1.18 ± 0.02	1400	273.19 ± 3.77	235.45 ± 5.16
post-guard	6450	1.17 ± 0.01	1.27 ± 0.02	2242	256.21 ± 2.89	203.19 ± 4.37

The location had a strong effect on foraging trip duration (RIW = 0.9) and on body mass change (RIW = 1) in all breeding stages, except for foraging trip duration in the guard stage (tables 2 and 3). Season and sex had very weak to no effect on foraging trip duration in all breeding stages, except in the pre-laying stage, where they both had a strong effect (tables 2 and 3). By contrast, season and sex had a strong effect on body mass change in all breeding stages, except for in the post-guard stage where season had a moderate effect. There was no effect of age on foraging trip duration or body mass change (tables 2 and 3). RIW was not calculated for the pre-laying stage, given that a single model explained all the variability of the response variable (table 3).

**Table 2.** Model selection explaining the variation in the response variables, foraging trip duration (days) and body mass change per day (g), for each breeding stage. The full model for all tests was: Response ∼ Season + Sex + Age + Location + (1| Penguin ID), but the model for foraging trip duration during the incubation stage also had the interaction term 'Location: Foraging trip duration' included to avoid violation of test assumptions. The best fit models presented had the sum of Akaike weights ≥ 0.9.

response variable	breeding stage	best fit models	Akaike weight (%)
foraging trip duration (days)	pre-laying	foraging trip duration ∼ location + season + sex	97
	incubation	foraging trip duration ∼ location + location: foraging trip duration	54
		foraging trip duration ∼ location + sex + location: foraging trip duration	20
		foraging trip duration ∼ location + season + location: foraging trip duration	20
	guard	null	30
		foraging trip duration ∼ location	18
		foraging trip duration ∼ sex	15
		foraging trip duration ∼ season	11
		foraging trip duration ∼ location + sex	9
		foraging trip duration ∼ location + season	7
	post-guard	foraging trip duration ∼ location + sex	35
		foraging trip duration ∼ location	27
		foraging trip duration ∼ location + season + sex	15
		foraging trip duration ∼ location + season	12
		foraging trip duration ∼ sex	4
body mass change per day (g)	pre-laying	body mass change ∼ location + season + sex	99
	incubation	body mass change ∼ location + season + sex	98
	guard	body mass change ∼ location + season + sex	76
		body mass change ∼ location + sex	12
		body mass change ∼ location + season	11
	post-guard	body mass change ∼ location + season + sex	83
		body mass change ∼ location + sex	15

**Table 3.** Relative importance weights (RIW) of the predictor variables for the best fit models obtained through model selection. The response variables are foraging trip duration (days) and body mass change per day (g) and the models are for different breeding stages. *RIW* ≥ *0.9* *=* *strong effect, 0.6* ≥ *moderate effect < 0.9, 0.5* ≥ *very weak effect* *<* *0.6, RIW* *<* *0.5* *=* *no effect.* For interactions, *RIW* *>* *0.7* *=* *strong effect and RIW* *>* *0.5* *=* *moderate effect.* NA indicates that the predictor variable was not included in the model for that breeding stage.

predictor variable	foraging trip duration			body mass change	
	incubation	guard	post-guard	guard	post-guard
location	0.9	0.3	0.9	1	1
season	0.2	0.2	0.3	0.9	0.8
sex	0.2	0.2	0.5	0.9	1
age	0	0	0	0	0
location: foraging trip duration	0.9	NA	NA	NA	NA

**Figure 5.**
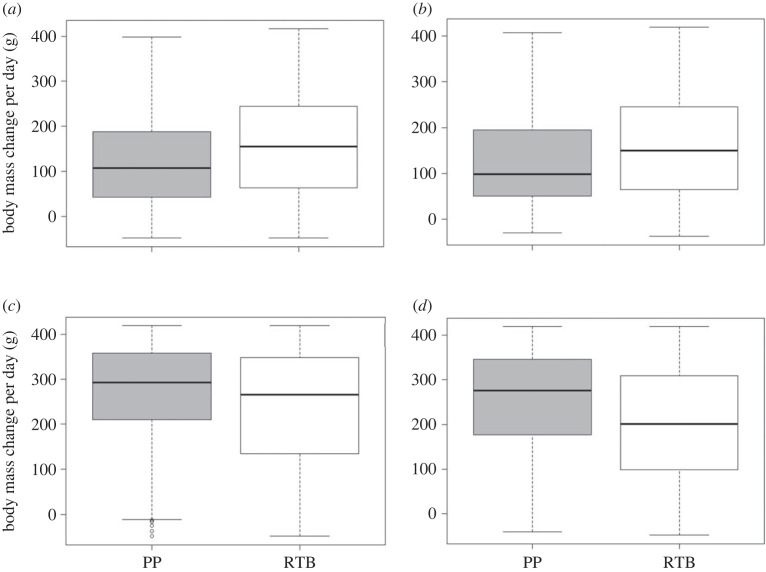
Boxplots showing the range, median and upper and lower quartiles for body mass change per day for the birds at each location for the 2015 and 2016 breeding seasons for the (*a*) pre-laying, (*b*) incubation, (*c*) guard and (*d*) post-guard breeding stages (Grey = Penguin Parade (PP), White = Radio-tracking Bay (RTB)). Outliers are indicated by open circles. (Refer to Table 5b for significance values).

In the pre-laying stage, birds at Penguin Parade had a significantly longer foraging trip duration and significantly lower body mass gain than at Radio-tracking Bay. A significantly lower body mass gain for birds at Penguin Parade also occurred in the incubation stage (tables 1 and 4, A1; figures 5, A1; refer to electronic supplementary material, table A1 and figure A1 in the supplementary material). However, this pattern was reversed in the guard and post-guard breeding stages. In the post-guard breeding stage, the birds at Radio-tracking Bay showed significantly longer foraging trip durations and significantly lower body mass gain than those at Penguin Parade. A significantly lower body mass gain for the birds at Radio-tracking Bay also occurred in the guard stage (tables 1 and 4, A1; Figures 5, A1).

**Table 4.** The statistics output showing the effect of location on (*a*) foraging trip duration for the mixed effects cox proportional hazards survival analysis and (*b*) body mass change for the linear mixed effects model in each breeding stage from the 2015 and 2016 breeding seasons. Statistical significance was accepted at a *p*-value < 0.05.

*A*	*z*-value	d.f.	*p*-value
pre-laying	2.70	35.89	<0.05
incubation	0.00	6.01	1.00
guard	−0.36	58.69	0.72
post-guard	−2.72	110.21	<0.05
*B*	*t*-value	d.f.	*p*-value
pre-laying	3.113	93.9239	<0.05
incubation	3.134	140.0869	<0.05
guard	−4.973	109.6576	<0.05
post-guard	−6.850	103.6000	<0.05

### 3.2. Reproductive success, mean laying date and sea surface temperature

**Figure 6.**
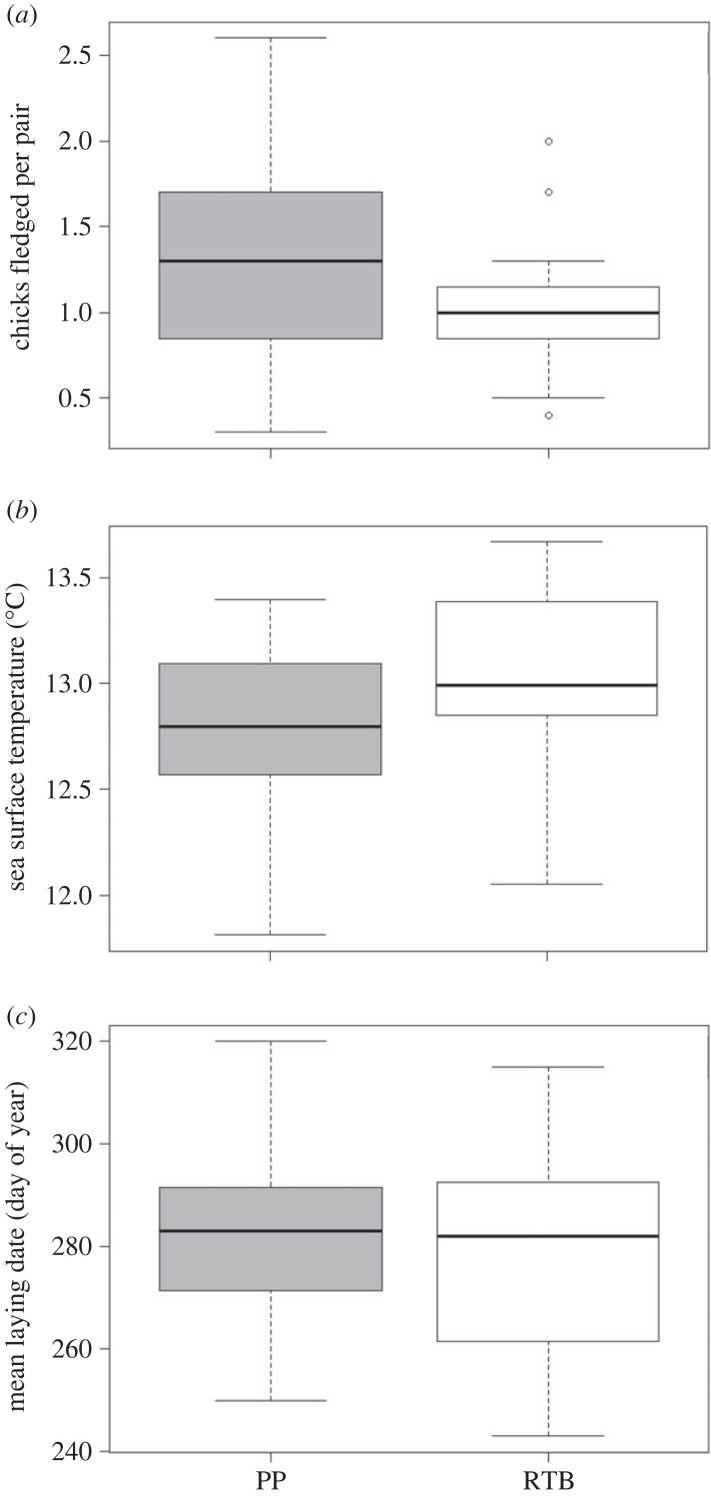
Boxplots showing the range, median, and upper and lower quartiles for (*a*) the number of chicks fledged per adult pair for the birds at each location (*t*-value = 3.0603, d.f. = 14, *p*-value < 0.05), (*b*) the sea surface temperature (°C) at the foraging sites of the birds from each location (*t*-value = −7.4567, d.f. = 14, *p*-value < 0.05) and (*c*) the mean egg-laying date (day of year) for the birds at each location (*t*-value = 2.5544, d.f. = 14, *p*-value < 0.05) for each breeding season from 2004–2018 (Grey = Penguin Parade (PP), White = Radio-tracking Bay (RTB)). Outliers are indicated by open circles.

**Figure 7.**
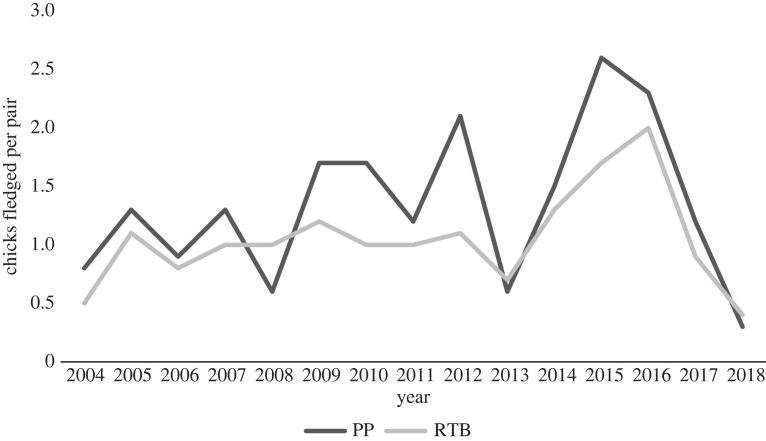
A line chart showing the average number of chicks fledged per adult pair for each breeding season from 2004–2018 for the birds at Penguin Parade and Radio-tracking Bay (Dark Grey = Penguin Parade (PP), Light Grey = Radio-tracking Bay (RTB)). The data for chicks fledged per pair from 2004–2015 was sourced from Sánchez *et al*. [43]).

**Figure 8.**
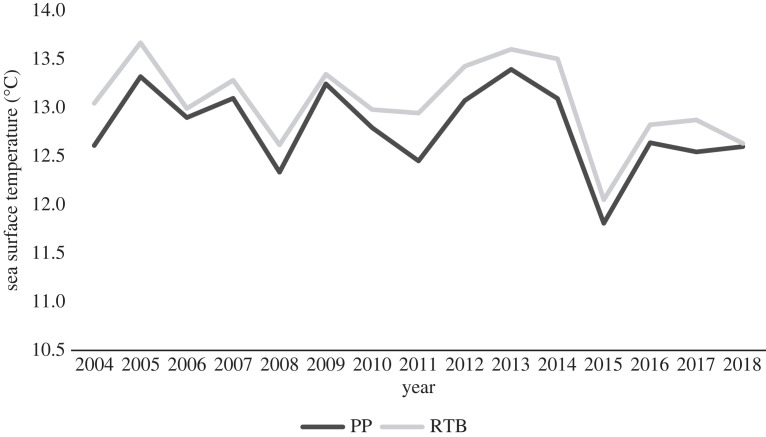
The average sea surface temperature (°C) for each breeding season from 2004–2018 for the foraging sites of the birds from each location, Penguin Parade and Radio-tracking Bay (Dark Grey = Penguin Parade (PP), Light Grey = Radio-tracking Bay (RTB)). Sea surface temperature is calculated as the average daily temperature between the 6th and 7th week prior to the mean egg-laying date for the birds at Penguin Parade for that year.

**Figure 9.**
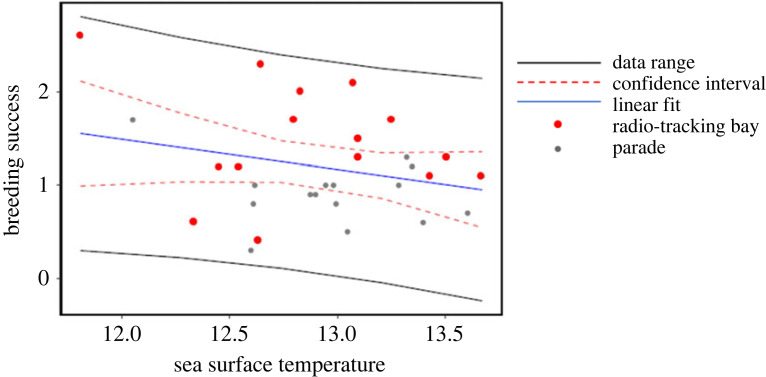
Decrease of breeding success as the sea surface temperature increases in the foraging range of two sub-colonies of little penguins at Phillip Island, Australia: Radio-tracking Bay and Parade from 2004 to 2018. Note that the Radio-tracking Bay (red dot) has a higher temperature than the Parade (grey dot). The graph shows the data range (black line), linear fit (blue line) and 95% confidence interval (red dotted line).

**Figure 10.**
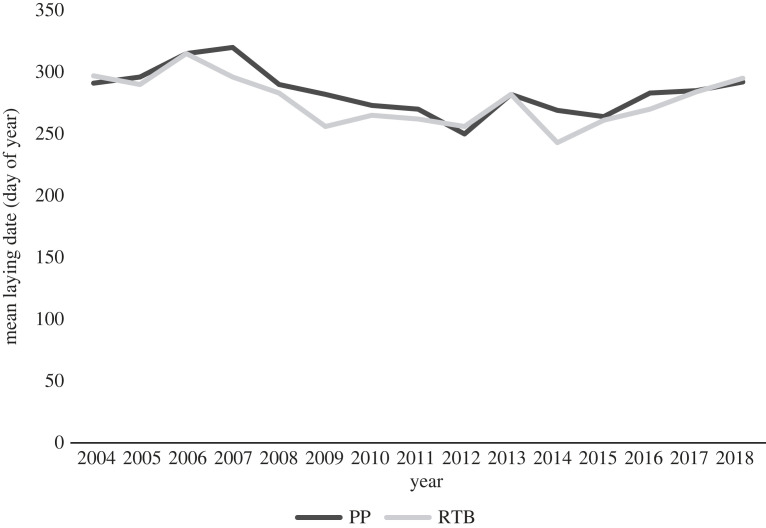
The mean egg-laying date (day of year) for each breeding season from 2004–2018 for the birds at each location, Penguin Parade and Radio-tracking Bay (Dark Grey = Penguin Parade (PP), Light Grey = Radio-tracking Bay (RTB)).

On average, 30% (0.3 ± 0.10 chicks) more chicks fledged per pair for the subcolony at Penguin Parade when compared with the subcolony at Radio-tracking Bay in the breeding seasons from 2004 to 2018 (*t*-value = 3.0603, d.f. = 14, *p*-value < 0.05) (electronic supplementary material, table A2; figures 6 and 7; refer to electronic supplementary material, table A2 in the supplementary material). Sea surface temperature was significantly higher in the foraging area of birds from Radio-tracking Bay than the foraging area of those from Penguin Parade over the same period (*t*-value = −7.4567, d.f. = 14, *p*-value < 0.05, figure 9) (electronic supplementary material, table A3; figures 6 and 8; refer to electronic supplementary material, table A3 in the supplementary material). Sea surface temperature also showed a negative relationship with each subcolony's reproductive success data (figure 9). There was also a significantly earlier mean egg-laying date for the subcolony at Radio-tracking Bay than at Penguin Parade (*t*-value = 2.5544, d.f. = 14, *p*-value < 0.05) (electronic supplementary material, table A4; figures 6 and 10; refer to electronic supplementary material, table A4 in the supplementary material).

## 4. Discussion

Our study revealed differences in foraging trip duration, body mass change, reproductive success and mean egg-laying date between the little penguin subcolonies. Penguins from the Penguin Parade subcolony took longer foraging trips. They gained less body mass per day than those from the Radio-tracking Bay subcolony during the pre-laying and incubation stages. However, this pattern was reversed between the subcolonies in the guard and post-guard breeding stages. The foraging zones of each subcolony also differed in SST where the foraging area of the birds from Radio-tracking Bay had a significantly higher SST than that of the birds from Penguin Parade. In both subcolonies, the reproductive success decreased with an increased sea surface temperature. Our results show differences in foraging and reproductive success between subcolonies, and differences in the environmental foraging conditions for penguins at each site.

Foraging trip duration and body mass change differed between subcolonies in all breeding stages, except for foraging trip duration in the guard stage. Foraging success depends on prey abundance and distribution [43]. The little penguins in the two subcolonies may have had different success in finding food because they foraged in different areas [43]. Birds may follow favourable prey conditions, so they travel to other areas depending on foraging opportunities [45,62]. Poor prey availability can cause birds to spend more time at sea to find enough food, changing the length of foraging trips and body mass [34]. Foraging differences due to season, sex, and age are observed in a range of seabird species [63–66]. However, in our study, the location of foraging and breeding site had a stronger influence on foraging trip duration and body mass change than the season, sex, and age, showing that different foraging and breeding sites strongly influence foraging success.

Accessibility to other foraging areas under the different breeding stages’ restrictions may result in differences in foraging success, even at this fine scale. The birds from Radio-tracking Bay may travel to more profitable feeding grounds in Port Phillip Bay during the less constrained pre-laying and incubation stages [7,43]. The birds from Penguin Parade are unlikely to travel to Port Phillip Bay, foraging instead in less productive offshore waters [43]. As a result, in the pre-laying and incubation stages, the Penguin Parade subcolony had a lower foraging success than the Radio-tracking Bay subcolony. By contrast, during the guard and post-guard breeding stages, birds at Radio-tracking Bay had a lower foraging success than the birds at Penguin Parade, suggesting that the local foraging grounds (less than 20 km from their breeding site) for the subcolony at Radio-tracking Bay had a lower prey availability than local foraging grounds for the subcolony at Penguin Parade. A more robust explanation, however, may be related to the bathymetry in the foraging areas [43,46,67]. The foraging area for the birds at Penguin Parade is shallower than the foraging area at Radio-tracking Bay. It may explain why the subcolony at Penguin Parade had a higher foraging success than the subcolony at Radio-tracking Bay during chick-rearing which has a higher constraint and fewer options for travelling further [43]. No effect of location on foraging trip duration was observed in the guard stage as birds only make one-day trips at this stage [8,47,68]. No effect was observed in the incubation stage as penguins travel longer distances to avoid local segregation and exploit food resources that will not be reached at chick rearing stages [69].

We detected variations in the success of different little penguin subcolonies on Phillip Island where the subcolony at Radio-tracking Bay had a lower foraging and reproductive success than the subcolony at Penguin Parade. The differing environments in each foraging zone may explain the variation in success. Birds at Radio-tracking Bay could shift to better foraging grounds used by birds at Penguin Parade. However, what keeps the subcolonies separated is the high level of foraging and nest competition that initially caused the segregation [13,15,16]. Although central place foraging seabird species, such as albatross and petrels, can have longer foraging ranges resulting in lower levels of competition than little penguins, these species are still constrained to the central place and are limited in their foraging range [70,71]. Therefore, this study's differences in foraging at the sub-colony level can apply to a wide range of central place foraging seabird species.

We observed differences in SST between the foraging areas of the two sub-colonies, even though they were not too far apart. The foraging area of the birds at Radiotracking Bay had a higher SST than the foraging area of the birds from the Penguin Parade. This could be due to differences in currents, wind or stratification and suggests differences in prey abundance and prey distribution at this small spatial scale [33,61,72]. It may explain the differences in foraging success and reproductive success within the colony. For little penguins elsewhere, a lower SST resulted in a higher probability of foraging in the area and a higher prey capture success [33]. One of the little penguins' preferred prey, sardines (*Sardinops sagax*), also shows these temperature patterns. Lower SST resulted in higher commercial sardine catch, implying a higher abundance of sardines clustered in areas with a lower SST [23]. A lower SST at the foraging site of the birds from Penguin Parade suggests that there may be a higher prey abundance and better foraging opportunities at this foraging site than at the foraging site of the birds from the Radio-tracking Bay subcolony. As a result, this difference in SST may have caused a higher number of chicks fledged per pair and thus a higher reproductive success for the birds at the Penguin Parade subcolony than the birds at the Radio-tracking Bay subcolony.

On average, birds at Radio-tracking Bay laid eggs earlier in the year than at Penguin Parade. The timing of egg-laying has been associated with the fat reserves of the penguins, primary productivity and SST [24,50,73]. An increase in temperature in early spring triggers the onset of breeding for little penguins at Phillip Island, with the mean laying dates occurring seven weeks after the SST increase, coinciding with the peak of marine productivity (chlorophyll-a concentration) [24], with similar patterns for little penguins in New Zealand [25]. Therefore, the warmer SST at Radio-tracking Bay may result in higher productivity earlier in the year, so the subcolony breeds a week earlier on average due to improved conditions. However, a high SST can limit productivity. This could explain why a higher SST at Radio-tracking Bay and earlier laying dates were also associated with lower reproductive success [26,27]. A similar pattern was found in little penguins near Perth, Western Australia [74].

## 5. Conclusion

Understanding how environmental conditions affect foraging and reproductive success between subcolonies of other seabird species can help develop and improve appropriate management strategies for conserving a range of seabird species that are colonial central place foragers. Such strategies could include the management of commercial fisheries that may compete with seabird prey resources in the different foraging areas within a colony and the increased protection of areas where subcolonies have low foraging and reproductive success.

Our study shows fine-scale variation within a colony due to high intra-specific competition. We found differences in foraging and reproductive success between subcolonies, likely due to differences in environmental conditions and prey availability in the foraging areas of each subcolony. Overall, our results show how seabirds can respond to small-scale changes in the environment and show the importance of investigating seabird species at the subcolony level to gain fine-scale knowledge that can be incorporated into conservation strategies for a range of colonial central place foraging seabird species.

## Data Availability

The raw data and the code used to analyse the data can be found in electronic supplementary material [75].
